# Recent Advances in the Molecular Genetics of Familial Hypertrophic Cardiomyopathy in South Asian Descendants

**DOI:** 10.3389/fphys.2016.00499

**Published:** 2016-10-28

**Authors:** Jessica Kraker, Shiv Kumar Viswanathan, Ralph Knöll, Sakthivel Sadayappan

**Affiliations:** ^1^Department of Internal Medicine, Heart, Lung and Vascular Institute, Division of Cardiovascular Health and Sciences, University of Cincinnati College of MedicineCincinnati, OH, USA; ^2^AstraZeneca R&D Mölndal, Innovative Medicines and Early Development, Cardiovascular and Metabolic Diseases iMedMölndal, Sweden; ^3^Integrated Cardio Metabolic Centre, Karolinska Institutet, Myocardial Genetics, Karolinska University Hospital in HuddingeHuddinge, Sweden

**Keywords:** hypertrophic cardiomyopathy, South Asians, β-myosin heavy chain, MYH7, cardiac myosin binding protein-C, MYPBC3

## Abstract

The South Asian population, numbered at 1.8 billion, is estimated to comprise around 20% of the global population and 1% of the American population, and has one of the highest rates of cardiovascular disease. While South Asians show increased classical risk factors for developing heart failure, the role of population-specific genetic risk factors has not yet been examined for this group. Hypertrophic cardiomyopathy (HCM) is one of the major cardiac genetic disorders among South Asians, leading to contractile dysfunction, heart failure, and sudden cardiac death. This disease displays autosomal dominant inheritance, and it is associated with a large number of variants in both sarcomeric and non-sarcomeric proteins. The South Asians, a population with large ethnic diversity, potentially carries region-specific polymorphisms. There is high variability in disease penetrance and phenotypic expression of variants associated with HCM. Thus, extensive studies are required to decipher pathogenicity and the physiological mechanisms of these variants, as well as the contribution of modifier genes and environmental factors to disease phenotypes. Conducting genotype-phenotype correlation studies will lead to improved understanding of HCM and, consequently, improved treatment options for this high-risk population. The objective of this review is to report the history of cardiovascular disease and HCM in South Asians, present previously published pathogenic variants, and introduce current efforts to study HCM using induced pluripotent stem cell-derived cardiomyocytes, next-generation sequencing, and gene editing technologies. The authors ultimately hope that this review will stimulate further research, drive novel discoveries, and contribute to the development of personalized medicine with the aim of expanding therapeutic strategies for HCM.

## The cardiovascular disease dilemma in south asian americans

**Figure 1:**
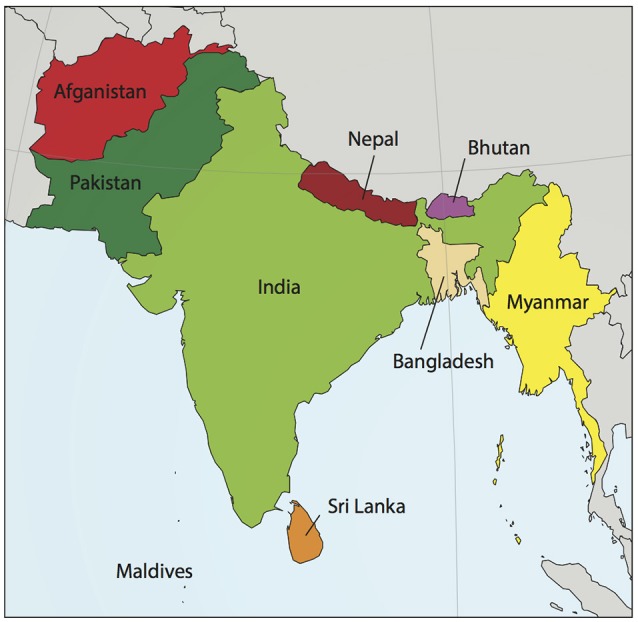
**Map of South Asia**. South Asia, sometimes referred to as the Indian subcontinent, includes the modern day countries of Afghanistan, Bangladesh, Bhutan, India, Nepal, the Maldives, Pakistan, and Sri Lanka. Myanmar, shown in the figure, is usually not included in South Asia except for population studies by the United Nations. Map modified from wikimedia commons South_Asia_(ed)update.PNG. An estimated 1.8 billion people live in this region, comprising one-fifth of the world's population and three-fifths of the global cardiovascular disease burden.

Although most cardiovascular disease (CVD) is preventable (Yusuf et al., 2004), it is the leading cause of death worldwide. The burden of CVD mortality is greatest in lower-income countries, yet is responsible for 1 in 4 deaths in the United States (Gupta et al., 2016). The South Asian subcontinent contains 20% of the world's population, yet accounts for about 60% of CVD worldwide. A map of this region, which includes the modern-day countries of Afghanistan, Bangladesh, Bhutan, India, Nepal, the Maldives, Pakistan, and Sri Lanka (Hajra et al., 2013), is shown in Figure 1. An estimated 1.8 billion people live in the South Asian region, and comprise one-fifth of the world's population. South Asians (SA) have a significantly increased risk of CVD when compared to their European counterparts (Anand et al., 2000; Yusuf et al., 2004; Fernando et al., 2015). Numerous studies have tried to account for this, assessing both traditional and non-traditional risk factors with inconsistent results (Chaturvedi, 2003; Palaniappan et al., 2010; Fernando et al., 2015). When considered together, risk factors alone do not account for the fact that SA immigrant populations are 3–5 times more likely to die of CVD than other ethnic groups (Gupta et al., 2006).

To date, the 2006 National Health Interview Survey is the only nationally representative data for obesity, CVD, and diabetes in Asian Americans (Barnes et al., 2008). However, the survey is limited in scope by self-reported collection measures, small sample size, and lack of long-term data (Mohanty et al., 2005; Narayan et al., 2010; Holland et al., 2011). Another glaring problem, especially when considering the existing interethnic comparative CVD studies, is the grouping of this extremely diverse population into the broad category of “Asian & Pacific Islander.” Genetic, environmental, and behavioral differences exist among ethnic subpopulations of this group, and such differences contribute to differential health outcomes (Graham et al., 2006). More accurate epidemiology data and risk models could be generated if these groups were disaggregated (Mohanty et al., 2005; Narayan et al., 2010; Holland et al., 2011; Gopal and Usher-Smith, 2016).

Within the last 40 years, changes in immigration policies have paved the way for dramatic migration of SA into the United States. SA were the fastest growing ethnic group in the United States between the years 2000 and 2010 (Gezmu et al., 2014). Currently, there are an estimated 3.5 million SA living in America, meaning that they constitute around 1% of the American population (Tang et al., 2012). Since its inception in 2010, the Mediators of Atherosclerosis in South Asians Living in America (**MASALA**) Study has examined cardiometabolic risk and CVD outcomes in South Asian Americans on a longitudinal basis (Kanaya et al., 2013). This groundbreaking study will undoubtedly generate comprehensive, population-specific data for this at-risk population. A pilot study completed in 2015, called the South Asian Heart Lifestyle Intervention (**SAHELI**) Study, initiated efforts to deliver culturally sensitive educational material to underserved SA at risk for CVD. However, despite these recent efforts health disparities continue to exist for this American subpopulation (Fernando et al., 2015). The paucity of cardiac disease data for Asian Americans is a barrier to addressing one of the most concerning public health issues of our time.

## Hypertrophic cardiomyopathy, a treatable form of CVD

### The cardiomyopathies

**Figure 2:**
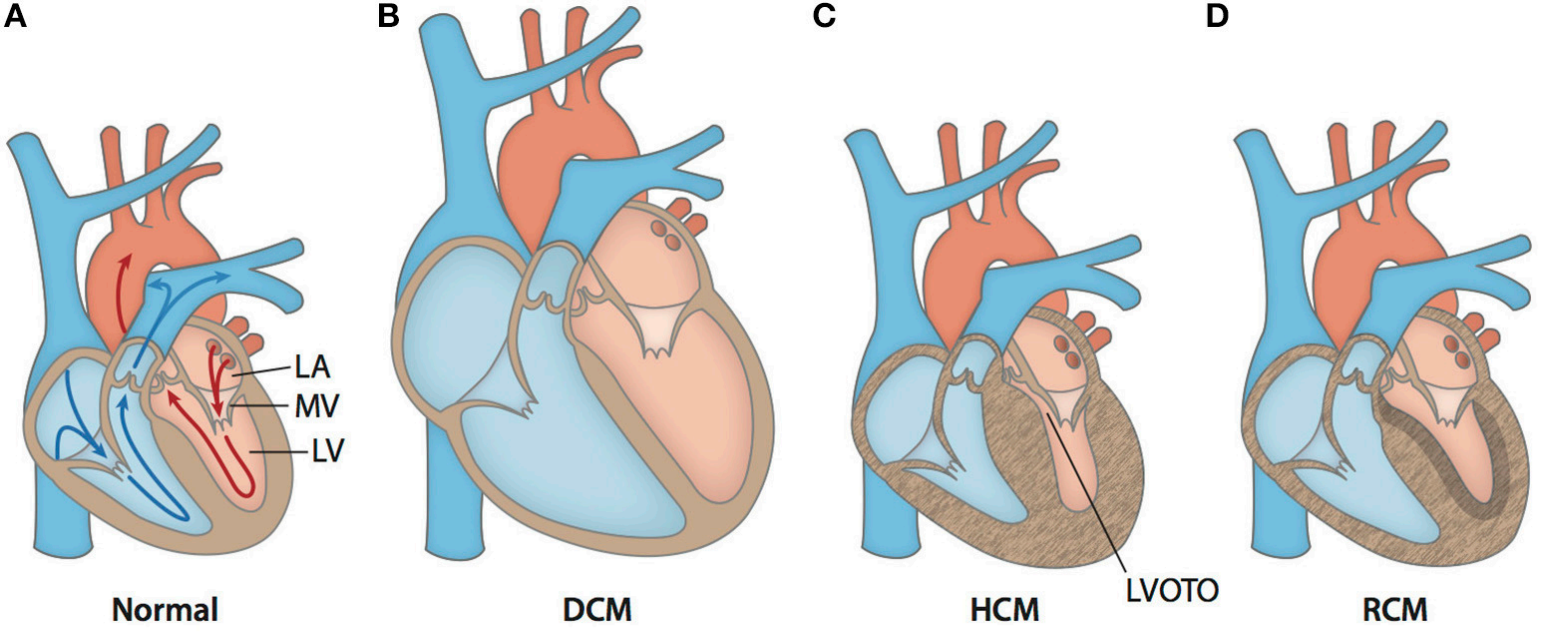
**Anatomical cross-sections of hearts with various forms of cardiomyopathy. (A)** Arrows outline the path of blood flow in a healthy heart. Blue corresponds to the deoxygenated venous return to the heart. Red corresponds to the oxygenated blood in the left side of the heart. After oxygen saturation in the lungs, blood is directed from the left atrium (LA) through the mitral valve (MV) into the left ventricle (LV). Then, it is pumped through the aorta to the rest of the body. **(B)** A typical enlarged heart seen in DCM, with thin and stretched walls and large chamber volumes. **(C)** A typical heart seen in HCM, with generalized hypertrophy and reduced size of the LV chamber. Increased wall thickness of the interventricular septum and protrusion of the MV into the outflow path contribute to left ventricular outflow tract obstruction (LVOTO). **(D)** A typical heart seen in RCM with ventricular walls of normally thickness. The walls are stiffened by increased fibrotic deposition, which is signified by darker shading.

Cardiomyopathies are a heterogeneous group of diseases of the myocardium associated with mechanical and/or electrical dysfunction. They usually exhibit inappropriate ventricular hypertrophy or dilatation and are due to a variety of causes that are commonly genetic (Elliott et al., 2008). Cardiomyopathies either are confined to the heart or are part of generalized systemic disorders, often leading to cardiovascular death or progressive heart failure-related disability (Maron et al., 2006). Cardiomyopathies are present in all populations, with ethnicity, age, and gender affecting disease severity and expression (McNally et al., 2015). The three main types of cardiomyopathy affecting the left ventricle are hypertrophic cardiomyopathy (HCM), dilated cardiomyopathy (DCM), and restrictive cardiomyopathy (RCM) (Elliott et al., 2008). Prototypical cases of HCM show abnormally large and misaligned myocytes localized to the interventricular septum and increased fibrosis (Gersh et al., 2011). The thickened and stiff ventricle reduces the compliance of the heart muscle, decreases preload, and contributes to diastolic heart failure (Jacoby et al., 2013; Hensley et al., 2015). On the other end of the spectrum, typical DCM cases show chamber volume dilatation and thin walls, which reduces contractile force, and causes systolic heart failure. In RCM, which is the least common of the cardiomyopathies, patients typically have normal wall thickness (Elliott et al., 2008; Gersh et al., 2011). A diagnosis of RCM is made when left ventricular pressure is pathologically increased at normal or reduced chamber volumes due to excessive fibrotic accumulation. The structural differences between a normal heart and those affected by DCM, HCM, or RCM are illustrated in Figure 2. Although the typifying cases of cardiomyopathies are clearly differentiated, it should be noted that the clinical presentation of end stage cardiomyopathy can overlap significantly.

### Overview of familial HCM

Familial HCM is the most common inherited cardiac disease and affects 1 in every 500 people worldwide (Maron et al., 2014). Since its first description in the 1950s, much progress has been made in elucidating the extremely heterogeneous genetic, morphogenic, and clinical profile of the disease (Elliott and McKenna, 2009). Symptoms of HCM are variable in severity and overlap with those of general CVD, including chest pain, shortness of breath, lightheadedness, palpitations, fatigue, and inability to perform vigorous exercise (Gersh et al., 2011). Another devastating manifestation of HCM is sudden cardiac death (SCD). Extensive left ventricular hypertrophy (LVH) in the absence of another cause can confirm a diagnosis of HCM (Brouwer et al., 2011). Non-parallel cell arrangement results in a characteristic “whirling” pattern and likely contributes to arrhythmogenicity in HCM patients. These histological changes are typically accompanied by increased collagen deposition and hyperplasia of microvasculature, pathologically narrowing the lumen and leading to symptoms related to myocardial ischemia (Shirani et al., 2000). HCM is typically classified as obstructive or non-obstructive depending on chamber anatomy, which determines the area through which blood can flow out of the heart. Obstructive HCM is normally associated with a worse prognosis due to the combined effects of systolic and diastolic dysfunction (Maron et al., 2003). Left ventricular outflow tract (LVOT) obstruction can result from a severely hypertrophied interventricular septum and/or abnormal mitral valve morphology (Nagueh and Mahmarian, 2006), as seen in Figure 2. The dynamic nature of LVOT obstruction aids in differentiation of HCM from other conditions in which LVH is the result of chronic and fixed pressure overload (Jacoby et al., 2013). LVOT obstruction, as well as other diagnostic hallmarks of HCM, can be detected via non-invasive imaging techniques such as echocardiography and cardiac MRI (Hensley et al., 2015) and in some cases provoked by altering loading conditions of the heart.

### Treatment of HCM

As is typical for many forms of CVD, many current therapeutic strategies for HCM try to alleviate symptoms and prevent complications. Although once considered rare and terminal, HCM has now emerged as a very treatable form of heart disease (Maron et al., 2014). Due to the variety of available surgical, pharmacological, electrical treatment options, HCM mortality rates have dropped to 0.5% per year (Maron et al., 2015). Beta-blockers and calcium channel blockers are used to improve diastolic function in patients with HCM (Brouwer et al., 2011; Hensley et al., 2015). Implantable cardiac defibrillators in conjunction with anticoagulants control potentially life-threatening arrhythmias and avoid SCD. Two common surgical procedures performed in about 3% of obstructive HCM patients are septal myectomy and alcohol septal ablation (Roma-Rodrigues and Fernandes, 2014). Both techniques are very successful at relieving LVOT obstruction and heart failure symptoms and have excellent long-term prognoses (Gersh et al., 2011; Jacoby et al., 2013; Maron et al., 2014). Early diagnosis and treatment of HCM is key to preventing long-term chamber remodeling and reducing the number of patients that advance to end-stage heart failure.

## Population genetics of HCM in south asians

### Genetic susceptibility of south asians

Modern humans first evolved in Africa 200,000 years ago and dispersed about 100,000 years thereafter (Majumder, 2010). Although details of the initial waves of people leaving Africa have been debated, archeological and genetic evidence points to a single South Asian dispersal route (Kivisild et al., 2003; Mellars, 2006; Majumder, 2010). From there, people populated the rest of Eurasia and then the world. Thus, using European or East Asian populations, who are genetic descendants of South Asians, to predict risk for their genetic ancestors is, by definition, an inappropriate model. The strong cultural ties of SA in America, and the tradition of consanguineous marriage offer unique opportunities to study genetics and allele frequencies of this population (Waldmüller et al., 2003). In particular, India's history of founder events predicts a high rate of recessive disease (Reich et al., 2009) and could act as a model for studying how genetic variation affects disease expression. Prioritization of South Asian Americans for screening of such recessive disease is a largely unexplored opportunity for researchers and clinicians alike. This also requires that such studies be carried out specifically in SA genomes, and not in comparison with Caucasian or East Asian genomes that share very few of these region-specific variants.

### Genetic heterogeneity of HCM

Recent advances in genetic sequencing techniques and the potential of therapeutic intervention in families with inherited cardiomyopathies have garnered this group of disorders much attention in the scientific community. Commercially available genetic screens are available to supplement a clinical diagnosis, and they have a mutation detection rate of 50–60% (Golbus et al., 2012). Genetic testing is recommended for at-risk family members who may also harbor a “private” familial variant (Gersh et al., 2011; Das et al., 2014). Once a variant is found, however, interpretation of its pathogenicity can be complicated. For example, distinguishing pathogenic variants from rare non-pathogenic variants or variants of unknown significance has proven difficult (Roma-Rodrigues and Fernandes, 2014). Currently, researchers have more variants of unknown significance than they can examine (Das et al., 2014). It would be impractical to study rare variants clinically, but recent large-scale sequencing projects have allowed statistical analysis of these variants (Lopes et al., 2013b). Typically, pathogenicity is estimated via *in silico* predictive algorithms that estimate a mutation's effect on protein structure and function and assess it in terms of evolutionary conservation (Ritchie and Flicek, 2014; Richards et al., 2015). While the use of multiple software programs to assess pathogenicity or a bioinformatics “pipeline” is recommended (Rehm et al., 2013), such programs still lack clinical data as input, making them insufficient for diagnostic purposes. Continued re-evaluation of all variants, even those previously considered benign, is necessary as predictive testing techniques continue to advance (Das et al., 2014; Richards et al., 2015). Family segregation studies and cellular/animal models are also options for studying pathogenicity of rare variants, but they are time-consuming and expensive.

### Necessity of population-specific genetic data for HCM

Apart from rare private familial mutations, ethnic “background variations” also affect penetrance and expressivity of genetic variants, further complicating genotype-phenotype predictions (Pan et al., 2012; McNally et al., 2015). Population-specific data have been reported in online databases of sequence variants, including those found in the NCBI, 1000 Genomes Project, and Exome Aggregation Consortium (ExAC). Allele frequencies of sarcomeric genes encoding β-myosin heavy chain, myosin binding protein C, and titin have been shown to differ among distinct ethnic groups (Golbus et al., 2012). Importantly, this study also reported that pathogenic variation in these genes was significantly higher than expected in the 1000 Genomes Database. This underscores the need for more comprehensive sequencing and screening of at-risk populations.

Population genetics studies on SA have been scarce, despite a well-documented increased risk of heart disease (Anand et al., 2000; Yusuf et al., 2004; Fernando et al., 2015). Southeast Asian genetic diversity is significantly underrepresented in the 1000 Genomes Project (Lu and Xu, 2013). Chambers et al. provided the first comprehensive genetic study of SA, performing whole-genome sequencing of 168 subjects (Chambers et al., 2014). Prior to the publication of this study in 2014, a mere two genomes had been used as references for the entire South Asian population, which complicated predictions of disease susceptibility (Kitzman et al., 2011; Gupta et al., 2012). In a recent study, the Exome Aggregation Consortium cited the underrepresentation of certain populations in previous genomic databases, notably Latinos and SA (Lek et al., 2016). Certain South Asian specific polymorphisms have been studied, but are of limited use due to small sample sizes that have very little statistical power (Dodani et al., 2012; Yadav et al., 2013). Extraction of meaningful information from raw datasets in online databases and association with clinical presentation is undoubtedly a necessary next step in the development of personalized medicine (Lek et al., 2016). It seems fitting that more genetic studies should be geared toward SA in order to study inherited treatable diseases like HCM. GenomeAsia 100k is a non-profit consortium aiming to generate Asian-specific genomic data. Importantly, the initial phase includes sequencing 10,000 reference genomes for all major ethnic groups within the Asian umbrella. As the name suggests, their ultimate goal is to generate genetic, microbiomic, clinical, and phenotypic data for 100,000 Asian genomes. If successfully completed, this genetic data will hugely contribute to future research and clinical efforts.

## Molecular genetics of HCM

**Figure 3:**
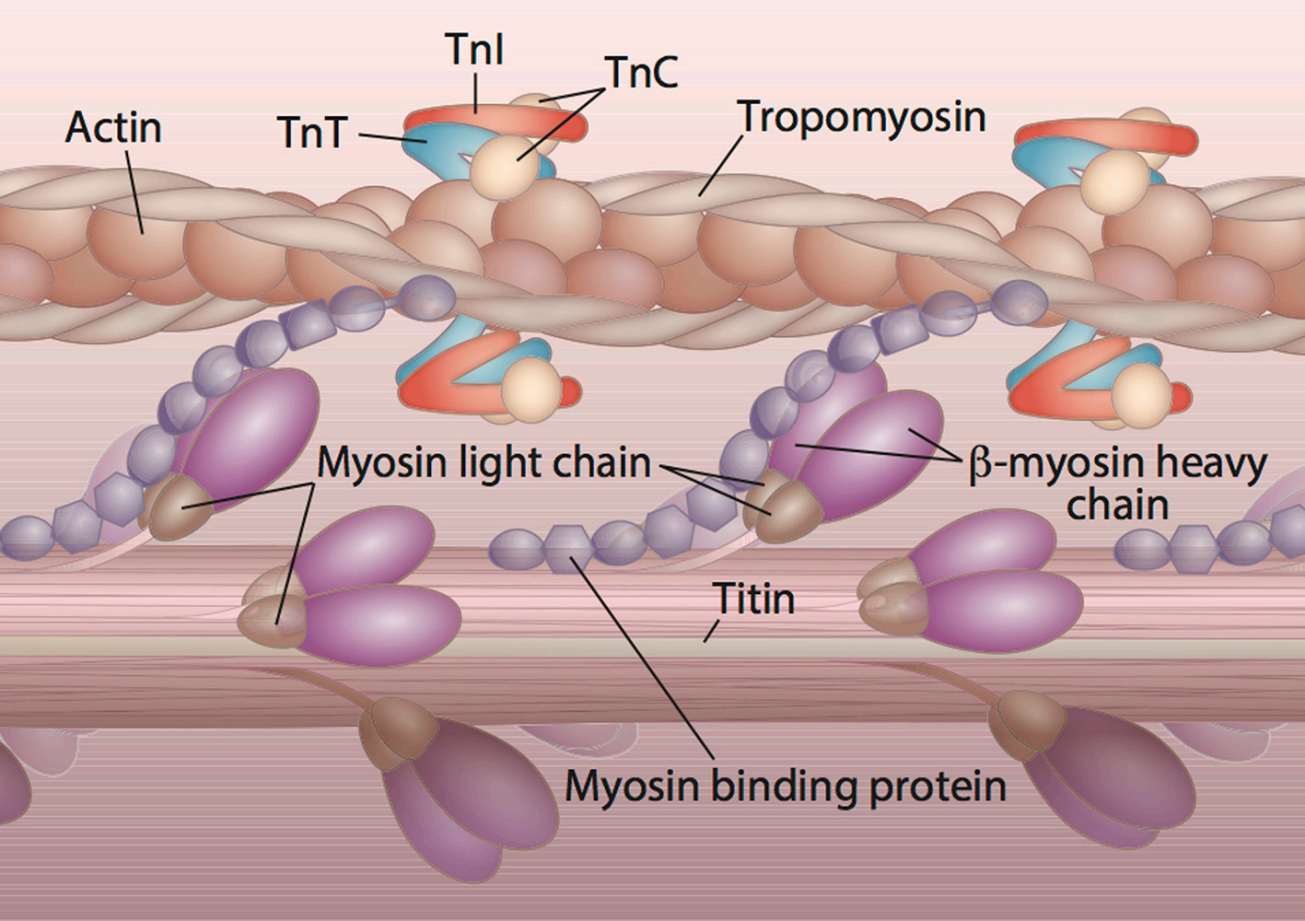
**Protein constituents and arrangement of the cardiac sarcomere**. The majority of HCM cases are the result of abnormal sarcomeric proteins. The sarcomere is the functional unit of striated muscle. The visible striations or bands of the cardiac sarcomere are due to the overlap of thin and thick filaments. The main constituents of the thick filament are β-myosin heavy chain, myosin binding protein C, titin, and the regulatory and essential light chains. Troponin T (TnT), troponin I (TnI), troponin C (TnC), tropomyosin, and actin comprise the thin filament of the sarcomere. Titin is a giant protein that connects the thick filament to the edge of the sarcomere and creates passive tension in resting muscle.

HCM is inherited in an autosomal dominant manner, and most of the 1400+ variants associated with HCM encode sarcomeric proteins (Brouwer et al., 2011; Schlossarek et al., 2011). Nine sarcomeric genes carry the majority of HCM-related mutations and encode the proteins: β-myosin heavy chain (*MYH7*), cardiac myosin binding protein C (*MYBPC3*), cardiac troponin T (*TNNT2*), cardiac troponin I (*TNNI3*), α-tropomyosin (*TPM1*), regulatory myosin light chain (*MYL2*), essential myosin light chain (*MYL3*), cardiac α-actin (*ACTC*), and cardiac troponin C (*TNNC1*). The arrangement and interaction of proteins in the cardiac sarcomere is illustrated in Figure 3. Another group of mutations can be found in the genes encoding sarcomeric Z-disc proteins such as muscle LIM protein, α-actinin, or telethonin (Knöll et al., 2010), but these are outside the scope of this review. The number of studies focusing on HCM-associated mutations in SA is disproportionately small when considering the size of this population and collective increased risk of CVD. To date, only 21 of the published variants associated with HCM have been documented in SA. These variants are listed in Table 1. In following with the Pareto principle (also known as the 80–20 rule), the majority of adverse HCM events comes from only a few central causes. In the case of HCM, over 80% of known HCM-associated mutations occur in *MYBPC3* and *MYH7* alone, while an additional 10% come from *TNNT2* and *TNNI3* (McNally et al., 2015). Thus, at least 90% of known HCM cases originate from four sarcomeric genes. The involvement of these four genes in the development of HCM is discussed below.

**Table 1:** **Sarcomeric gene variants associated with HCM in South Asians**.

**Gene**	**Variant**	**Pathogenicity status**	**References**
**MYH7**			
	c.2221G > T Arg712Leu	N/A	Sakthivel et al., 2000; Waldmüller et al., 2003
	ΔE927	N/A	Waldmüller et al., 2003
	NM_000257.3(MYH7):c.2609G > A (p.Arg870His) rs36211715	Pathogenic/Likely pathogenic	Tanjore et al., 2006; Bashyam et al., 2007
	NM_000257.3(MYH7):c.1544T > C (p.Met515Thr) rs863224900	Likely pathogenic	Rai et al., 2009
	NM_000257.3(MYH7):c.1816G > A (p.Val606Met) rs121913627	Pathogenic / Likely pathogenic	Rai et al., 2009
	NM_000257.3(MYH7):c.2146G > A (p.Gly716Arg) rs121913638	Conflicting interpretations of pathogenicity	Rai et al., 2009
	NM_000257.3(MYH7):c.2686G > A (p.Asp896Asn) rs606231340	Pathogenic	Rani et al., 2014; Selvi Rani et al., 2015
	I524K	N/A	Selvi Rani et al., 2015
**MYBPC3**			
	NM_000256.3(MYBPC3):c.3641G > A (p.Trp1214Ter) rs730880597	Pathogenic	Bashyam et al., 2012
	D570fs	N/A	Tanjore et al., 2008
	NM_000256.3 (MYBPC3):c.2308G > A (p.Asp770Asn) rs36211723	Pathogenic/Likely pathogenic	Tanjore et al., 2008
	NM_000256.3 (MYBPC3):c.3815-1G > A rs397516044	Pathogenic/Likely pathogenic	Waldmüller et al., 2003; Tanjore et al., 2008; Dhandapany et al., 2009; Srivastava et al., 2011; Kuster et al., 2015
	NM_000256.3 (MYBPC3):c.1357_1358delCC (p.Pro453Cysfs) rs727503203	Pathogenic	Waldmüller et al., 2003
**TNNT2**			
	NM_001001430.2 (TNNT2):c.430C >T (p.Arg144Trp) rs483352832	Conflicting interpretations of pathogenicity	Rani et al., 2012a, 2014
	g.5857 A28V	N/A	Rani et al., 2012a
	NM_001001430.2 (TNNT2):c.318C > G (p.Ile106Met) rs3729547	Uncertain significance	Rani et al., 2012a
**TNNI3**			
	g. 2601 C > G P82R	N/A	Rani et al., 2012b
	g. 4019 G > A R98Q	N/A	Rani et al., 2012b; Ramachandran et al., 2013
	NM_000363.4 (TNNI3):c.422G > A (p.Arg141Gln) rs397516347	Pathogenic/Likely pathogenic	Rani et al., 2012b; Ramachandran et al., 2013
	NM_000363.4 (TNNI3):c.485G > A (p.Arg162Gln) rs397516354	Pathogenic/Likely pathogenic	Rani et al., 2012b; Ramachandran et al., 2013
**TPM1**			
	NM_001018005.1 (TPM1):c.644C > T (p.Ser215Leu) rs199476316	Conflicting interpretations of pathogenicity	Gupte et al., 2015; Rangaraju et al., 2012; Selvi Rani et al., 2015

PubMed published variants have been listed with Human Genome Variation Society (HGVS) nomenclature (noted as NM_XXXX), Single Nucleotide Polymorphism Database (dbSNP) Reference SNP cluster ID (noted as rsXXXX), and ClinVar pathogenicity status where possible. For published variants without a ClinVar reference, N/A is listed under pathogenicity status. Intronic variants and those classified as benign or likely benign have not been included in the table. None of these mutations were reported to be coexistent with other mutations that could confound pathogenicity status of the listed variant.

## Thick filament mutations associated with HCM

The thick filament of the sarcomere consists of bundles of myosin molecules, which form a cylindrical backbone as seen in Figure 3. The bipolar head domains project radially to interact with the thin filament during contraction (Spudich, 2014). Although attractive, the idea that myosin mutations causing hypercontractility lead to HCM and those causing hypocontractility lead to DCM has not been definitively established (Spudich, 2014). However, changes in calcium sensitivity (higher in HCM, decreased in DCM) have been discussed, and a direct link between calcium sensitivity, which induces heart growth via tension generation, and HCM has recently been established (Davis et al., 2016).

### *MYH7* mutations

*MYH7* encodes cardiac β-myosin heavy chain (β-MHC), which is the major part of the thick filament of the sarcomere. The intrinsic ATPase activity of the catalytic domains of β-MHC provides the energy for sarcomeric contraction. Missense mutations in the globular head domain of β-MHC tend to produce HCM because they prevent interaction with actin, which is necessary for proper sarcomeric contraction (Volkmann et al., 2007). The lever-like myosin converter domain and globular head region show significant genetic constraint, and mutations in these regions are associated with severe forms of HCM (Homburger et al., 2016). The combination of normal and mutant or “poison peptide” causes detrimental structural and functional effects in the sarcomere (Brouwer et al., 2011). To date, 289 mutations have been found in *MYH7* that are thought to produce HCM, and this gene comprises 40% of the genetic profile of the disease (Morimoto, 2008; Stenson et al., 2014). Only eight of these variants have been published in SA. Mutations in *MYH7* are associated with early-onset and extensive LVH (Roma-Rodrigues and Fernandes, 2014), and clinically are associated with an increased risk of atrial fibrillation, SCD and heart failure (Wang et al., 2008; Lopes et al., 2013a). In the Indian population, it has been predicted that the frequency of *MYH7* mutations may be lower than their *MYBPC3* counterpart, although validation in a large sample is required for confirmation (Morimoto, 2008).

Due to its clinical severity, the *MYH7-R403Q* variant was the first HCM-causing mutation to be discovered (Geisterfer-Lowrance et al., 1990; Nag et al., 2015). This mutation, which affects the “cardiomyopathy loop” at the interface of actin and myosin (Marian, 2002; Volkmann et al., 2007) is associated with severe LVH and nearly 100% penetrance of SCD at a young age (Lopes et al., 2013a). Studies on this variant have been particularly helpful in studying both loss-of-function and gain-of-function mutations associated with HCM. Loss-of-function mutations result in reduced contractile force (Nag et al., 2015), while gain-of-function mutations generate supraphysiological force (Seidman and Seidman, 2001; Moore et al., 2012). The diverse manifestations of the same mutation on sarcomere biomechanics emphasizes the need to fully characterize the signaling pathways involved in disease development and develop specialized therapies.

### *MYBPC3* mutations

*MYBPC3* encodes the cardiac isoform of myosin binding protein C (cMyBP-C). cMyBP-C is a thick filament-associated protein that acts as a tether for the myosin head domain and also associates with actin and titin. cMyBP-C is found in the C-zone of the sarcomere and has both structural and regulatory roles in sarcomere assembly. Mutations in *MYBPC3* typically take longer to manifest than those in *MYH7*, presenting in middle age or later and after the typical reproductive age (Wang et al., 2008). To date, around 346 *MYPBC3* mutations have been found that are associated with HCM, and comprise at least 40% of the genetic profile of the disease (Morimoto, 2008; Stenson et al., 2014). Of these 346, a mere 5 have been published in South Asian subjects. As opposed to *MYH7* mutations, the majority of those affecting *MYBPC3* result in abnormal truncation of the C-terminus of cMyBP-C (Kuster and Sadayappan, 2014). The degradation of these aberrant expression products is thought to contribute to HCM via haploinsufficiency, i.e., inadequate amounts of the normal protein product (Brouwer et al., 2011).

Perhaps the most well-cited example of a region-specific polymorphism in SA is a 25-bp deletion in *MYBPC3 (MYBPC3*^Δ*Int*32^) (Waldmüller et al., 2003; Dhandapany et al., 2009). The genetic heterogeneity of HCM, background genetic noise, and cost of sequencing make screening impractical for the general population (Kapplinger et al., 2014). However, the prevalence of the *MYBPC3*^Δ*Int*32^ variant is at least 4% in SA but virtually non-existent in Caucasians. The exclusivity of this allele to South Asian descendants makes genetic screening effective and practical for this population (Kuster and Sadayappan, 2014). The 25-bp deletion from intron 32 of *MYBPC3* causes a reading frameshift in translation, and the aberrant protein product cMyBP-C^C10mut^ lacks exon 33 and includes abnormal parts of exon 34 and part of the 3′ untranslated region (Waldmüller et al., 2003; Dhandapany et al., 2009). The altered C10 domain cannot normally link cMyBP-C to the myosin heavy chain and leads to contractile dysfunction (Kuster et al., 2015). Double heterozygotes, or patients carrying both the *MYBPC3*^Δ*Int*32^ mutant allele and another mutant allele in *MYH7-E927del*, seem to have a more severe HCM phenotype suggesting an additive effect of these mutations (Waldmüller et al., 2003). Systematic studies are currently underway at various laboratories to determine if the *MYBPC3*^Δ*Int*32^ variant is sufficient to cause HCM.

## Thin filament mutations associated with HCM

α-tropomyosin, along with the troponins T, I, and C form the troponin-tropomyosin complex that regulates contraction in striated muscle. As seen in Figure 3, this complex is interdigitated with actin and winds together to form the thin filament of the sarcomere. This regulatory complex prevents strong association between the thick and thin filament in low Ca^2+^ and ATP conditions (McNally et al., 2015). Thin filament mutations typically increase Ca^2+^ sensitivity of tension development (Ashrafian et al., 2011) or uncouple Ca^2+^ sensitivity from phosphorylation (Papadaki et al., 2015).

### *TNNT2* mutations

The connection between *TNNT2*, the gene encoding the cardiac isoform of troponin T (TnT), and HCM was first reported in 1994 (Thierfelder et al., 1994). *TNNT2* variants account for about 5% of HCM cases (Morimoto, 2008). TnT mutants have been linked to increased risk of SCD at a young age (Marian, 2002; Lopes et al., 2013a) but typically patients carrying these variants exhibit little to no hypertrophy (Chandra et al., 2005). TnT mutants are thought to alter cross-bridge kinetics, thereby limiting shortening velocity at maximal Ca^2+^ activation and increasing the energetic cost of contraction (Sweeney et al., 1998). In mice, degree of Ca^2+^ sensitization from mutations in TnT were found to directly correspond to arrhythmic risk and were reduced with the Ca^2+^ desensitizer Blebbistatin (Baudenbacher et al., 2008). To date, 43 HCM-related mutations in *TNNT2* have been documented in the general population (Morimoto, 2008; Stenson et al., 2014). In SA, 3 *TNNT2* variants have been published that are associated with HCM, as seen in Table 1.

### *TNNI*3 mutations

*TNNI3*, which encodes troponin I, was first linked to HCM in 1997 (Kimura et al., 1997). *TNNI3* mutations make up about 5% of the genetic profile of HCM (Morimoto, 2008). 38 HCM-related mutations in *TNNI3* are known, and 4 of these have been documented in SA (Stenson et al., 2014) as can be seen in Table 1. Recently, mutations in the highly conserved inhibitory peptide region of troponin I were found to alter myofilament Ca^2+^ sensitivity (Westfall et al., 2002). Mutations in this important regulatory region are thought to produce increased tension at physiological Ca^2+^ levels and prevent relaxation.

## Non-sarcomeric mutations associated with HCM

Non-sarcomeric genes are also involved in HCM, and some researchers have proposed that the disease should be reclassified into sarcomeric and non-sarcomeric because pathophysiology and prognosis differ significantly between these groupings (Olivotto et al., 2011; McNally et al., 2015). The variable penetrance and expressivity in HCM means that the same mutation may cause severe disease in one person, but a completely normal phenotype in another (Tanjore et al., 2010). Genotypically positive but phenotypically negative patients have perplexed researchers, and suggest a role of modifier genes in clinical outcomes (García-Honrubia et al., 2016).

This review will focus on a well-documented angiotensin-1 converting enzyme (*ACE*) polymorphism in SA that contributes to the HCM phenotype in a dose-dependent manner (Rai et al., 2008). The insertion allele (I) corresponds to the presence of a 287-bp transposable Alu element repeat, and the deletion allele (D) corresponds to the absence of this sequence from the *ACE* gene (Marian, 2002). LVH was most severe in patients with the DD genotype, which corresponds to higher plasma and tissue ACE levels. LVH was less pronounced in heterozygous ID individuals and least severe in II individuals, who were found to have less circulating ACE (Marian, 2002). Long-term activation of components in the renin-angiotensin-aldosterone system such as ACE is thought to lead to vascular remodeling and hypertrophy seen in HCM. However, significant inconsistencies have been reported regarding *ACE* I/D polymorphisms in HCM patients (Yang et al., 2013). To avoid limitations due to genetic heterogeneity and small sample size, large-scale studies or of *ACE* I/D polymorphisms in genetically distinct groups are necessary (Kolder et al., 2012; Luo et al., 2013).

## Secondary HCM can be a result of extracardiac disease

Patients with extracardiac disease can also present with LVH similar to that seen in HCM, and these are listed in Table 2. Many of these diseases present with other obvious systemic findings, allowing early differential diagnosis (Roma-Rodrigues and Fernandes, 2014). A notable exception is milder forms of Fabry disease, which has often been inaccurately diagnosed as HCM with the same prevalence as some sarcomeric mutations (Sachdev et al., 2002). This is significant because Fabry disease is treatable with enzyme replacement therapy, while the chamber remodeling associated with HCM is currently irreversible. For this reason, mutations in the genes causing Fabry disease and Danon disease are often included in genetic test panels for HCM. Of these systemic diseases, only 6 have been documented in SA, as shown in Table 2. This further underscores the need to note subjects' ancestry when performing genetic testing and the relevance of this information to the scientific community. The number of mitochondrial disorders causing phenocopies of HCM is too extensive for this review and have not been included in Table 2.

**Table 2:** **Systemic diseases that present with secondary HCM**.

**Type of disease**	**Name of disease**	**Gene**	**Gene product**	**References**
Lysosomal storage disease	Fabry disease	*GLA*	α-Galactosidase	**Wani et al., 2016**; Sachdev et al., 2002
Glycogen storage disease (GSD)	Pompe disease (GSD type II)	*GAA*	α-1,4-Glucosidase	Lim et al., 2014; **Van der Ploeg et al., 1989; Raju et al., 2010; Jegadeeswari et al., 2012**
Glycogen storage disease	Danon disease (GSD type IIb)	*LAMP2*	LAMP2	Endo et al., 2015
Glycogen storage disease	Cori's disease (GSD type IIIa)	*AGL*	Amylo-1,6-glucosidase	Lee et al., 1997; **Lucchiari et al., 2002**
Glycogen storage disease	Anderson's disease (GSD type IV)	*GBE1*	Amylo-1,4-1,6-transglucosidase	Aksu et al., 2012
Neuromuscular disorder	Friedreich's ataxia	*FXN*	Frataxin, mitochondrial protein	Kumari and Usdin, 2009
Neuromuscular disorder	Myotonic muscular dystrophy	*DMPK*	Myotonic dystrophy protein kinase	Lau et al., 2015
RASopathy	Noonan's syndrome	*RIT1*	Rit1	**Aggarwal et al., 2011**
RASopathy	LEOPARD syndrome (noonan syndrome with multiple lentigines)	*PTPN11*	SHP2	Jayaprasad and Madhavan, 2015
		*RAF1*	Protein tyrosine phosphatase	
		*BRAF*		
Metabolic storage disorder	Wolff-Parkinson-white syndrome with HCM	*PRKAG2*	γ2 regulatory subunit of AMP-activated protein kinase (AMPK)	**Sidhu and Roberts, 2003; Bashyam et al., 2012**
Lipodystrophy	Congenital generalized lipodystrophy (CGL) types 1–4 Berardinelli-Seip syndrome	1. AGPAT2 2. BSCL2 3. CAV1 4. PTRF	1.1-acylglycerol-3-phosphate O-acyltransferase 2 2. Seipin 3. Caveolin 4. Polymerase I, transcript release factor	Lupsa et al., 2010; **Rahman et al., 2013**
Mitochondrial disorders			Mitochondrial DNA	
	Various: MELAS, MERRF, CPEO, NARP, Leigh Syndrome, etc.			Finsterer and Kothari, 2014

Secondary HCM refers to hypertrophic cardiomyopathy that originates from another disease, the origin of which is outside of the heart. References in bold typeface correspond to PubMed published cases documented in South Asians.

## Molecular mechanisms underlying the development of HCM

On the sarcomeric level, several mechanisms are thought to link the genotype to the clinical phenotype of HCM. Increased myofilament Ca^2+^ sensitivity is believed to be pro-arrhythmogenic, although the exact molecular mechanism(s) involved remain(s) unclear. Altered Ca^2+^ handling, inefficient energy utilization, and increased mechanical stretch may play a role in the development of anatomical and functional changes observed in hearts with HCM (Huke and Knollmann, 2010; Ashrafian et al., 2011). Stabilization and prolongation of the Ca^2+^ transient would pathologically activate intracellular signaling pathways and diminish relaxation, contributing to electrical conduction abnormalities and diastolic dysfunction (Roma-Rodrigues and Fernandes, 2014). A predicted consequence of altered Ca^2+^ sensitivity is inefficient energy usage, including increased cross-bridge turnover, increased ATPase activity, and mitochondrial energetic abnormalities causing oxidative stress (Ashrafian et al., 2011; Brouwer et al., 2011). Energy and calcium are intrinsically linked to muscle mechanics due to excitation-contraction coupling; thus, force production of myofilaments will be abnormally increased as a result of Ca^2+^ sensitization. Therapeutic strategies are currently being explored which reinstate normal Ca^2+^ homeostasis, lower Ca^2+^ sensitivity, and increase efficiency of energy utilization (Brouwer et al., 2011). A recent breakthrough showed that increased Ca^2+^ sensitivity of troponin C causes prolonged myofilament tension, concentric sarcomeric addition, and hypertrophy consistent with HCM (Davis et al., 2016). DCM was linked to decreased Ca^2+^ sensitivity in the same study.

## Current efforts toward precision medicine for HCM

The current technologies outlined in this section are still in their infancy, and require much optimization in order to be developed into clinical therapies. However, the existence of HCM-associated variants unique to SA represents a largely unexplored opportunity to develop molecular models for cardiac disease. The vast size of this population makes application of these models to clinical treatments a logical and potentially lucrative next step.

### Reprogramming of cardiomyocytes

Adult somatic cells can be reprogrammed using sets of transcription factors to generate human induced pluripotent stem cells (iPSCs) and then functional cardiomyocytes (Kamdar et al., 2015), offering researchers the opportunity to study the molecular mechanisms of CVD and myocardial tissue regeneration following ischemic injury. iPSC-derived cardiomyocytes have been successfully used to study systemic diseases that present with HCM, including Pompe disease (Huang et al., 2011) and LEOPARD syndrome (Carvajal-Vergara et al., 2010).

The pathogenic effects of *MYH7* and *MYBPC3* mutations have been demonstrated using iPSC-derived cardiomyocytes (Ross et al., 2016). Lan and colleagues administered calcium blockers to iPSC-derived cardiomyocytes from HCM patients harboring the *MYH7-R663H* mutation (Lan et al., 2013). These drugs seemed to prevent structural and functional abnormalities typical of HCM, suggesting that their use in prophylaxis warrants investigation. Han and colleagues similarly examined the *MYH7-R442G* mutation (Han et al., 2014). They performed whole-transcriptome sequencing and documented an upregulation in gene expression in various cell proliferation signaling pathways including Wnt/β-catenin, Calcineurin-NFAT, and FGF that are thought to contribute to the disease phenotype. Tanaka and colleagues investigated how Endothelin-1, a circulating vasoconstrictive peptide, induced the HCM phenotype in iPSC-derived cardiomyocytes containing the *MYBPC3-G999-Q1004del* mutation (Tanaka et al., 2014). Lan et al. specified their proband's ethnicity as African-American, but the two studies assessing *MYH7* variants lack ethnicity information. *In vivo* direct cardiac reprogramming of somatic cells into cardiomyocytes is a potential offshoot of current reprogramming techniques but has not yet been tested in humans (Sadahiro et al., 2015). For HCM in particular, the possibility of converting cardiac fibroblasts into functional cardiomyocytes could theoretically ameliorate hypertrophy and improve diastolic function. As reprogramming technology advances, these techniques could offer a renewable source of cardiomyocytes and deliver medicine individually tailored to each patient (Chen and Qian, 2015; Tanaka et al., 2015).

### Variant databases

Next generation sequencing (NGS) protocols for screening cardiomyopathies have been successfully developed (Gómez et al., 2014), and they will continue to uncover new variants. Improved methods of distinguishing between benign and pathogenic mutations will enhance the accuracy of risk prediction of genetic screens for HCM (Kapplinger et al., 2014). Further, combination of gene expression analysis and clinical phenotypic assessments are necessary to uncover unknown modifier genes in HCM patients (Han et al., 2014). The development of online databases for HCM variants could aid physicians in the timing and implementation of electrical or surgical treatment options (McNally and Puckelwartz, 2015). As mentioned in the preceding sections, inclusion of ethnicity data in such databases has important genetic consequences and thus is clinically relevant. The future of personalized medicine will rely heavily on such collaborative efforts.

### Gene editing technology

Of the cardiovascular diseases, primary inherited cardiomyopathies such as HCM are perhaps the strongest candidates for gene editing technologies (Strong and Musunuru, 2016). Directed nucleases such as Transcription Activator-Like Effector Nucleases, Zinc Finger Nucleases (TALENs), and Clustered, Regularly Interspaced, Short Palindromic Repeats (CRISPR) and CRISPR-associated 9 (Cas9) systems allow for specific editing of individual gene mutations. This CRISPR/Cas9 system is the current frontrunner of these gene modification technologies and has the potential to facilitate causation studies, by both creating and correcting a specific mutation and documenting the associated phenotype (Han et al., 2014). This technology promises to provide researchers with more accurate model for studying cardiomyopathies, circumventing the inherent ethical issues when studying human subjects (Waddington et al., 2016). To date, the CRISPR/Cas9 system has been used to successfully engineer cardiomyopathy into in zebrafish and mice models, and is currently being applied to larger animals such as pigs and non-human primates (Duncker et al., 2015).

The CRISPR/Cas9 system has been used to edit the genomes of mice with Duchenne muscular dystrophy, an X-linked genetic disorder that presents with cardiomyopathy. The normal muscle phenotype in mice embryos was rescued following correction of the mutant dystrophin (*Dmd*) gene (Long et al., 2014). Considerable shortcomings of this technology include the variable correction efficiency and the introduction of mutagenesis at an incorrect but sequentially similar location (Strong and Musunuru, 2016). Currently there are no established techniques that can identify off-target mutagenesis. However, despite these concerns, CRISPR/Cas9-mediated genome editing is an exciting frontier in the genetic realm. In theory, it could eliminate inherited genetic disorders such as the cardiomyopathies altogether.

### Small-molecule inhibitors

The recently developed small myosin ATPase inhibitor MYK-461 was found to reduce sarcomeric contractility, LVH, myocyte disarray, and fibrosis in mice with HCM (Green et al., 2016). Although MYK-461 has huge potential as a therapeutic agent, it is not effective at reversing myocyte disarray and fibrosis once substantial hypertrophy has developed (Green et al., 2016). This underscores the need for expanded genetic screening in at-risk populations in order to administer earliest such preventative treatments. Pharmacologic treatments typically relieve heart failure symptoms without altering disease progression (Green et al., 2016). However, in a recent study, the calcium channel blocker Diltiazem used to treat hypertension and chest pain was shown to prevent reduction in left ventricular chamber size in HCM patients carrying *MYBPC3* mutations (Ho et al., 2015). This observed attenuation of HCM development is promising, and the drug could be used to treat at-risk family members before they notice the functional heart failure symptoms associated reduced cardiac output. Continued refinement of small-molecule inhibitors to arrest or even reverse hypertrophy, myocyte disarray, and fibrosis will likely aid in untangling the complex cellular mechanisms involved HCM development (Frey et al., 2012).

## Conclusions

The failure of risk factors to explain the increased prevalence of CVD in SA suggests a strong genetic causation that needs active characterization. For HCM in particular, it is imperative that SA receive higher priority for systematic genome-wide studies. Such studies will uncover population specific variants contributing to susceptibility, and could lead to better diagnosis and management of HCM. By taking advantage of novel technologies such as NGS, human iPSC derived cardiomyocytes, and gene editing nucleases, it is hoped that we will be able to significantly improve the lives of patients affected by genetic heart failure. It is essential that novel genomics technologies are used to reduce health disparities among marginalized and minority groups rather than perpetuate them (Rotimi and Jorde, 2010). Efforts such as those led by GenomeAsia100k will provide the necessary genetic data for future discovery.

Genetic variants, molecular mechanisms, and clinical phenotypes of HCM vary on a patient-by-patient basis. The Precision Medicine Initiative announced by President Obama in 2015 aims to personalize the current “one-size-fits-all” model of healthcare from the level of diagnosis all the way to delivery. In this article, we underscore the relevance of ethnicity data to genetic studies in particular, and stress that future studies specifically address the very large yet largely neglected South Asian population. The Sarcomeric Human Cardiomyopathy Registry (**SHaRe**) is an exciting interdisciplinary model that brings together geneticists and cardiologists to share and develop longitudinal, personalized data for patients with HCM and DCM. Although these efforts have mostly been undertaken in developed countries (Fox, 2015), this review has emphasized the need to address highly populated and underserved regions such as South Asia. Considering the genetic and clinical heterogeneity of the disease and its potential treatability, established cardiovascular centers of excellence provide the best possible treatment for HCM patients (Gersh et al., 2011; Maron et al., 2014). The Amrita Institute of Medical Sciences and Research in India has undertaken the task of becoming such a center, and if successful is expected to have dramatic impacts on HCM healthcare delivery in the South Asian subcontinent (Maron, 2015).

## Author contributions

JK, SV, RK, and SS designed and coordinated the proposed review article, analyzed literature data, and wrote the article. All authors discussed the work and commented on the manuscript.

### Conflict of interest statement

The authors declare that the research was conducted in the absence of any commercial or financial relationships that could be construed as a potential conflict of interest.
